# Effects of dietary macronutrients and body composition on glucose homeostasis in mice

**DOI:** 10.1093/nsr/nwaa177

**Published:** 2020-08-06

**Authors:** Sumei Hu, Jacques Togo, Lu Wang, Yingga Wu, Dengbao Yang, Yanchao Xu, Li Li, Baoguo Li, Min Li, Jianbo Li, Guanlin Wang, Xueying Zhang, Chaoqun Niu, Mohsen Mazidi, Alex Douglas, John R Speakman

**Affiliations:** State Key Laboratory of Molecular Developmental Biology, Institute of Genetics and Developmental Biology, Chinese Academy of Sciences, Beijing 100101, China; State Key Laboratory of Molecular Developmental Biology, Institute of Genetics and Developmental Biology, Chinese Academy of Sciences, Beijing 100101, China; University of Chinese Academy of Sciences, Beijing 100049, China; State Key Laboratory of Molecular Developmental Biology, Institute of Genetics and Developmental Biology, Chinese Academy of Sciences, Beijing 100101, China; University of Chinese Academy of Sciences, Beijing 100049, China; Institute of Biological and Environmental Sciences, University of Aberdeen, Aberdeen AB24 2TZ, UK; Key Laboratory of Molecular Pharmacology and Drug Evaluation (Ministry of Education of China), School of Pharmacy, Yantai University, Yantai 264005, China; State Key Laboratory of Molecular Developmental Biology, Institute of Genetics and Developmental Biology, Chinese Academy of Sciences, Beijing 100101, China; University of Chinese Academy of Sciences, Beijing 100049, China; Institute of Biological and Environmental Sciences, University of Aberdeen, Aberdeen AB24 2TZ, UK; State Key Laboratory of Molecular Developmental Biology, Institute of Genetics and Developmental Biology, Chinese Academy of Sciences, Beijing 100101, China; State Key Laboratory of Molecular Developmental Biology, Institute of Genetics and Developmental Biology, Chinese Academy of Sciences, Beijing 100101, China; State Key Laboratory of Molecular Developmental Biology, Institute of Genetics and Developmental Biology, Chinese Academy of Sciences, Beijing 100101, China; University of Chinese Academy of Sciences, Beijing 100049, China; State Key Laboratory of Molecular Developmental Biology, Institute of Genetics and Developmental Biology, Chinese Academy of Sciences, Beijing 100101, China; University of Chinese Academy of Sciences, Beijing 100049, China; State Key Laboratory of Molecular Developmental Biology, Institute of Genetics and Developmental Biology, Chinese Academy of Sciences, Beijing 100101, China; University of Chinese Academy of Sciences, Beijing 100049, China; Institute of Biological and Environmental Sciences, University of Aberdeen, Aberdeen AB24 2TZ, UK; School of Basic Medical Sciences, University of Dali, Dali 671000, China; State Key Laboratory of Molecular Developmental Biology, Institute of Genetics and Developmental Biology, Chinese Academy of Sciences, Beijing 100101, China; University of Chinese Academy of Sciences, Beijing 100049, China; Institute of Biological and Environmental Sciences, University of Aberdeen, Aberdeen AB24 2TZ, UK; State Key Laboratory of Molecular Developmental Biology, Institute of Genetics and Developmental Biology, Chinese Academy of Sciences, Beijing 100101, China; University of Chinese Academy of Sciences, Beijing 100049, China; Institute of Biological and Environmental Sciences, University of Aberdeen, Aberdeen AB24 2TZ, UK; State Key Laboratory of Molecular Developmental Biology, Institute of Genetics and Developmental Biology, Chinese Academy of Sciences, Beijing 100101, China; State Key Laboratory of Molecular Developmental Biology, Institute of Genetics and Developmental Biology, Chinese Academy of Sciences, Beijing 100101, China; Institute of Biological and Environmental Sciences, University of Aberdeen, Aberdeen AB24 2TZ, UK; State Key Laboratory of Molecular Developmental Biology, Institute of Genetics and Developmental Biology, Chinese Academy of Sciences, Beijing 100101, China; Institute of Biological and Environmental Sciences, University of Aberdeen, Aberdeen AB24 2TZ, UK; CAS Center for Excellence in Animal Evolution and Genetics (CCEAEG), Kunming 100101, China

**Keywords:** dietary macronutrients, body fat mass, fasting blood glucose, glucose tolerance, mice

## Abstract

As a major health issue, obesity is linked with elevated risk of type 2 diabetes. However, whether disrupted glucose homeostasis is due to altered body composition alone, or whether dietary macronutrients play an additional role, independent of their impact on body composition, remains unclear. We investigated the associations between macronutrients, body composition, blood hormones and glucose homeostasis. We fed C57BL/6N mice 29 different diets with variable macronutrients for 12 weeks. After 10 weeks, intraperitoneal glucose tolerance tests were performed. Generalized linear models were generated to evaluate the impacts of macronutrients, body composition and blood hormones on glucose homeostasis. The area under the glucose curve (AUC) was strongly associated with body fat mass, but not dietary macronutrients. AUC was significantly associated with fasting insulin levels. Six genes from transcriptomic analysis of epididymal white adipose tissue and subcutaneous white adipose tissue were significantly associated with AUC. These genes may encode secreted proteins that play important previously unanticipated roles in glucose homeostasis.

## Teaser

Hu et al. found that disrupted glucose homeostasis in obese mice is mainly due to high body fatness, and dietary fat plays an additional role on fasting glucose levels.

## INTRODUCTION

Obesity is one of the most serious global health issues. Excess body weight is the sixth most important risk factor contributing to the overall burden of diseases [1]. It increases the risk of many chronic diseases, including type 2 diabetes, cardiovascular diseases, hypertension, stroke and cancer [1]. Obesity is one of the main risk factors for impaired glucose homeostasis and type 2 diabetes [2], and is widely agreed to stem from prolonged energy imbalance [3]. A major contributor to this imbalance is excess energy intake rather than low energy expenditure [4].

Changing macronutrient composition in the diet may promote energy intake and therefore impact adiposity, however, there is still debate over whether high fat (HF), high sugar, low protein or all three are responsible for the elevated intake [5–8]. It is also uncertain to what extent disrupted glucose homeostasis, and other features of poor metabolic health in individuals with obesity, are a consequence of elevated body weight and adiposity alone, or whether macronutrient composition of the diet is an additional or the sole factor of importance.

Some evidence supports the idea that adiposity is not responsible by itself for the features of metabolic dysfunction. For example, there is a subgroup of people who have obesity yet are metabolically healthy, so called ‘fat yet fit’ individuals [9], and also individuals who are normal weight, but with dysfunctional metabolic status [10]. These individuals are possibly explained by differing dietary macronutrient compositions and energy intakes that underpin their weight status [9,10]. Moreover, in individuals that are overweight or with obesity as well as type 2 diabetes, glucose levels improve rapidly when energy intake is decreased, even before weight loss [11]. Glucose tolerance improves rapidly following bariatric surgery, also often in advance of significant weight loss, in both mice and humans [12,13]. Body weight loss and reduced energy intake can further improve metabolic syndrome and fatty liver, including improved insulin sensitivity, blood glucose and lipid control [14,15].

Some studies, focusing on specific diets with high/low protein, fat or carbohydrate, have reported impacts of different diets on glucose tolerance and fasting glucose/insulin levels in rodents [16–23]. Yet in many of these studies, only two or three diets were investigated in each study, and the different diets with variable compositions provided inconsistent results. More comprehensive dietary manipulations including larger numbers of diets have been performed [8,23] but these have not included glucose tolerance tests (GTTs). Moreover, in a previous study where a large dietary matrix was used and GTTs were performed, this study did not attempt to partition the effects of diet directly on the GTT from the impacts mediated via body weight [24]. It is consequently still unclear how macronutrients impact glucose homeostasis in animals, and whether the effects on glucose homeostasis are direct or mediated via body composition. Therefore, we designed 29 different diets varying orthogonally in their macronutrient compositions to separate different macronutrient effects, and then fed these diets to C57BL/6N mice for 12 weeks [8]. We previously explored the impact of these diets on food intake and body weight regulation [8]. In the present study the objective was to investigate the effects of dietary protein, fat and sucrose content on glucose homeostasis; specifically to explore the associations between dietary macronutrients, body fat mass, lean mass and fasting blood glucose (FBG) levels and the area under the curve (AUC) of the standard intraperitoneal glucose tolerance tests (ipGTT) using generalized linear modeling (GLM).

## RESULTS

### Dietary protein content does not affect fasting glucose levels and glucose tolerance in mice

Body mass and fat mass of the mice increased gradually during 12 weeks on the diets with variable protein content and either 60% or 20% fat (Fig. S1). The increase in body mass was mainly due to the increase in fat mass (Fig. S1). No significant difference was observed in the fat mass of the mice fed diets with variable protein content and either fixed 20% (ANOVA, F*_5, 52_ *= 1.891, *P* = 0.112) or 60% fat (ANOVA, F*_5, 53__ _*= 0.941, *P* = 0.463). There was no significant difference in lean mass between the mice fed diets with different protein contents, except significant differences between 5% and 20% protein at 60% fat (ANOVA, F*_5, 53__ _*= 2.573, *P* = 0.037), and between 5% and 25% protein at 20% fat (ANOVA, F*_5, 49__ _*= 3.005, *P* = 0.019).

**Figure 1.**
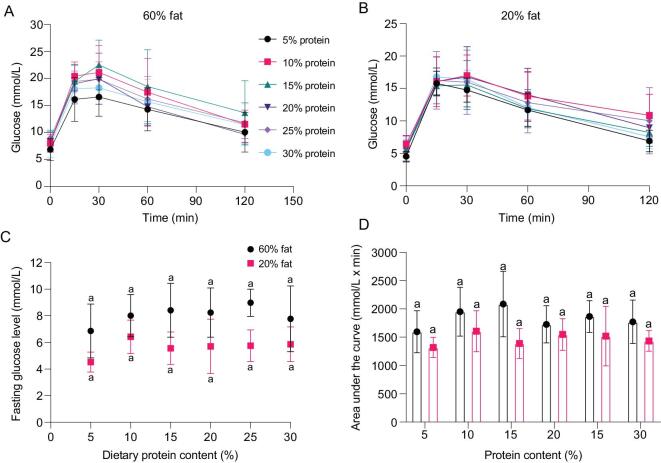
Glucose tolerance test of the mice fed on diets with fixed 60% fat or 20% fat and variant protein content. Ten mice per diet were used. Values were presented as mean ± SD. Glucose curve of the mice fed on diets with fixed 60% fat (A) or 20% fat (B) over 2 hours. (C) Fasting blood glucose levels. (D) Area under the glucose curve (AUC). See also Fig. S1.

IpGTT were performed to investigate the glucose tolerance in mice fed on diets with variable protein content. Fasting glucose levels were measured before glucose injection. There was no significant difference in FBG in mice fed diets with 60% fat (ANOVA, F*_5, 48__ _*= 1.283, *P* = 0.287), and only significantly lower FBG levels in the mice fed with 5% protein than 20% protein without any difference between other groups when dietary fat was fixed at 20% (ANOVA, F*_5, 50__ _*= 2.656, *P* = 0.033) (Fig. 1). AUC over the 2 h following glucose injection was not significantly different in the mice fed on diets with variable protein contents and either fixed 20% (ANOVA, F*_5, 55__ _*= 1.129, *P* = 0.356) or 60% fat (ANOVA, F*_5, 55__ _*= 1.802, *P* = 0.128) (Fig. 1). Overall these results demonstrated that dietary protein levels between 5% and 30% had no appreciable effect on glucose homeostasis.

### Dietary fat content affects fasting glucose levels and glucose tolerance in mice

Similarly, body mass, fat mass and lean mass of the mice all increased gradually in mice fed diets with variable fat content and fixed 10% or 25% protein content (Fig. S2). Increased body mass was mainly from increased body fat mass rather than lean mass (Fig. S2). In the mice fed the fixed 10% protein diets, body mass (ANOVA, F*_5, 52_* = 3.007, *P* = 0.019) and fat mass (ANOVA, F*_5, 52_* = 2.632, *P* = 0.034) were significantly different between the diets with 10% and 50% fat, but there were no differences among other diets with variable fat content. Significant differences were observed in body mass (ANOVA, F*_5, 52_* = 14.805, *P* = 5.054 × 10^−9^) and fat mass (ANOVA, F*_5, 56_* = 22.356, *P* = 3.011 × 10^−12^) in mice fed with variable fat and fixed 25% protein content after 10 weeks feeding. There were no significant differences in lean mass of all mice fed on diets either with 10% (ANOVA, F*_5, 52_* = 1.440, *P* = 0.226) or 25% dietary protein (ANOVA, F*_5, 54_* = 1.522, *P* = 0.199) (Fig. S2).

**Figure 2.**
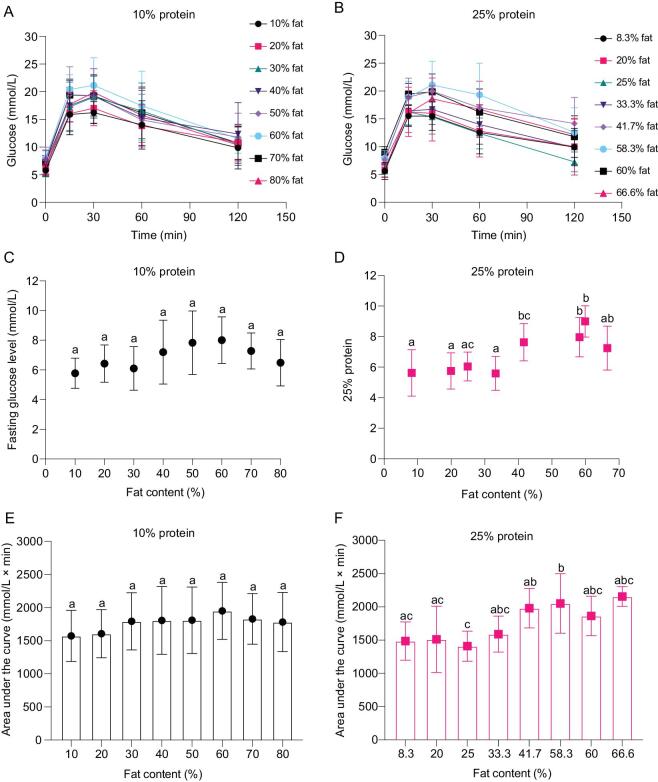
Glucose tolerance test of the mice fed on diets with fixed 10% protein or 25% protein and variant fat content. Nine to eleven mice per diet were used. Values were presented as mean ± SD. Glucose curve of the mice fed on diets with fixed 10% protein (A) or 25% protein (B) over 2 hours. (C) Fasting blood glucose levels of the mice on diets with 10% protein. (D) Fasting blood glucose levels of the mice on diets with 25% protein. (E) Area under the glucose curve (AUC) of the mice on diets with 10% protein. (F) AUC of the mice on diets with 25% protein. See also Fig. S2.

In the ipGTT, no significant difference was observed between any groups in both FBG levels (ANOVA, F*_7, 68_* = 2.310, *P* = 0.036, overall significance) and AUC of the glucose curve (ANOVA, F*_7, 70__ _*= 0.731, *P* = 0.646) of the mice fed on diets with fixed 10% protein and variable fat content (Fig. 2). Significantly different FBG levels (ANOVA, F*_7, 73_* = 10.429, *P* = 6.004 × 10^−9^) and AUC of the glucose curve (ANOVA, F*_7,75__ _*= 4.987, *P* = 1.13 × 10^−4^) were observed in the mice fed on diets with fixed 25% protein and variable fat content (Fig. 2). Both FBG levels and AUC of the glucose curve were significantly higher in the mice fed on diets with fixed 25% protein content and 41.7% or higher fat content, with no significant differences when fat content was between 8.3% and 33.3% (Fig. 2). The results suggest chronic exposure to high fat diets (more than 41.7% fat) impaired glucose tolerance.

### Dietary sucrose content does not impact glucose tolerance in mice

**Figure 3.**
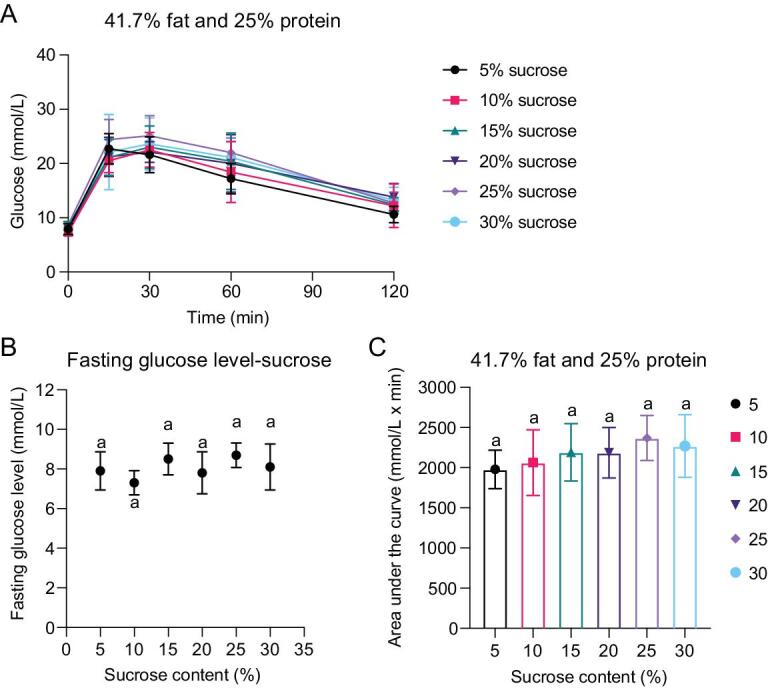
Glucose tolerance test of the mice fed on diets with fixed 25% protein and 41.7% fat and variant sucrose content. Five mice per diet were used. Values were presented as mean ± SD. (A) Glucose curve over 2 hours. (B) Fasting blood glucose levels. (C) Area under the glucose curve (AUC). See also Fig. S3.

To investigate the effect of dietary sucrose on glucose homeostasis, we then fixed both fat (41.7%) and protein (25%) and only changed sucrose content (5%–30%) in the diets. Body mass, fat mass and lean mass were all increased gradually during the experimental period in the mice fed on these diets (Fig. S3). Body mass was gained mainly from fat mass, consistent with the changes observed in the other 24 diets (Fig. S3). There was no significant difference in body mass (ANOVA, F*_5, 24__ _*= 0.407, *P* = 0.839), fat mass (ANOVA, F*_5, 24__ _*= 0.413, *P* = 0.835) and lean mass (ANOVA, F*_5, 24__ _*= 1.237, *P* = 0.323) between the mice fed on diets with different sucrose content after 10 weeks feeding. No difference was observed in either FBG levels (ANOVA, F*_5, 24__ _*= 1.567, *P* = 0.207) or AUC of the glucose curve (ANOVA, F*_5, 24__ _*= 0.867, *P* = 0.517) between the mice fed on diets with variable sucrose content (Fig. 3), demonstrating no extra effect of high sucrose (HS) on glucose tolerance.

### GLM analysis of AUC of the glucose curve and FBG levels

**Figure 4.**
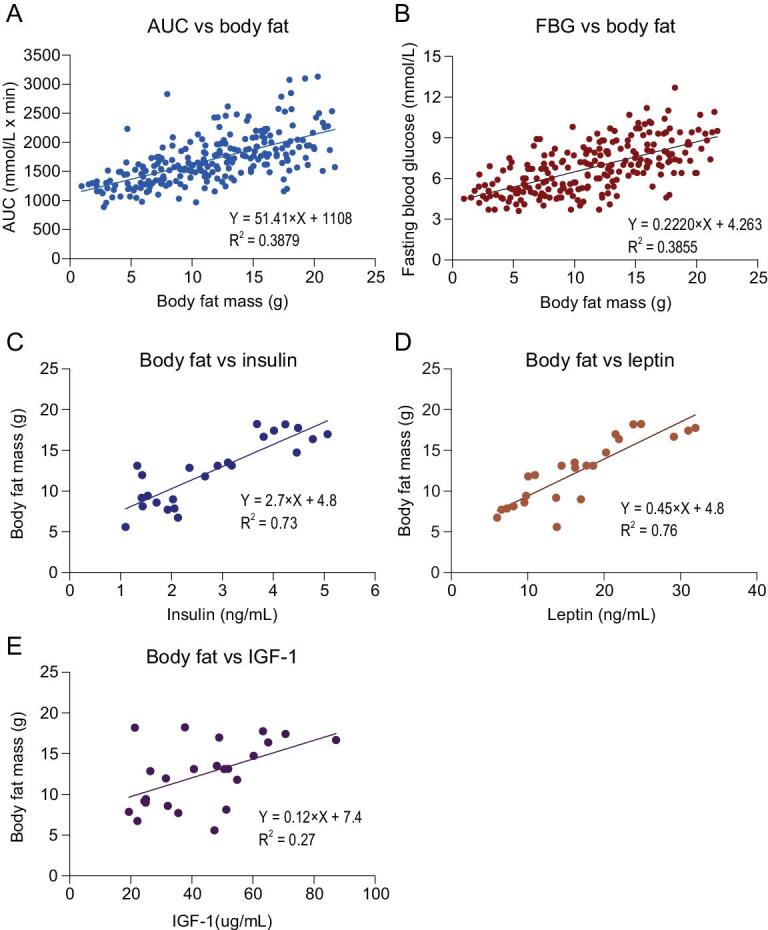
Linear regression between area under the glucose curve (AUC) or fasting blood glucose and body fat mass, or between body fat mass and blood hormone levels. Values were individual measurements for each mouse. (A) AUC. (B) Fasting blood glucose. (C) Insulin. (D) Leptin. (E) IGF-1. See also Fig. S4.

A GLM was performed to explore the association between the AUC and body fat mass, lean mass, dietary protein and fat contents. There were no significant interactions (*P* > 0.05) and no effect of lean mass, and therefore these variables were removed and a revised model generated (AUC-GLM1). In the revised model, body fat mass was significantly related to AUC (*P* < 0.0001). However, neither dietary protein (*P* = 0.335) nor fat (*P* = 0.666) content had effects on AUC of the glucose curve. Fat mass explained 38.8% of the variation in AUC across the individual mice (Fig. 4A).

Similarly, a GLM was also performed to explore the association between FBG and body fat mass, dietary protein and fat contents. Body fat mass, lean mass and dietary protein or fat all had no interaction effects (*P* > 0.05), and lean mass had no effect on the FBG levels (*P* = 0.880). Therefore, we revised the GLM by removing these non-significant terms. The final GLM (FBG-GLM1) demonstrated that both body fat mass (*P* < 0.0001) and dietary fat content (*P* = 0.003) were significantly related to FBG levels, but dietary protein content showed no significant association (*P* = 0.405). The model explained 40.1% of the variation in FBG levels, with body fat mass explaining 38.6% of the variation (Fig. 4B).

GLM by definition only detects linear effects, and non-linear relationships may in theory exist between the FBG levels or AUC and each of the co-variates. To examine if there were any non-linear effects between FBG levels or AUC against body fat mass, lean mass, dietary protein or fat, we analyzed the residuals from the GLMs, and plotted these residuals against body fat mass and dietary fat content (Fig. S4). The gradients of the linear regression models of the plots were −3.0 × 10^−15^ and 1.0 × 10^−14^, respectively, for AUC, and −1.0 × 10^−10^ and −3.0 × 10^−10^, respectively, for fasting glucose levels (*P* > 0.05 in all cases), suggesting there were no non-linear effects on either FBG levels or AUC.

As a separate experiment in the diets with variable sucrose content, a GLM was generated for both FBG and AUC of the glucose curve against body fat mass, lean mass and dietary sucrose content. None of these terms were significantly associated with either FBG levels or AUC of the glucose curve. There was also no potential non-linearity in the relationships (Fig. S5).

### Blood insulin levels were associated with both FBG levels and AUC of the glucose curve, but leptin was only associated with FBG levels

**Figure 5.**
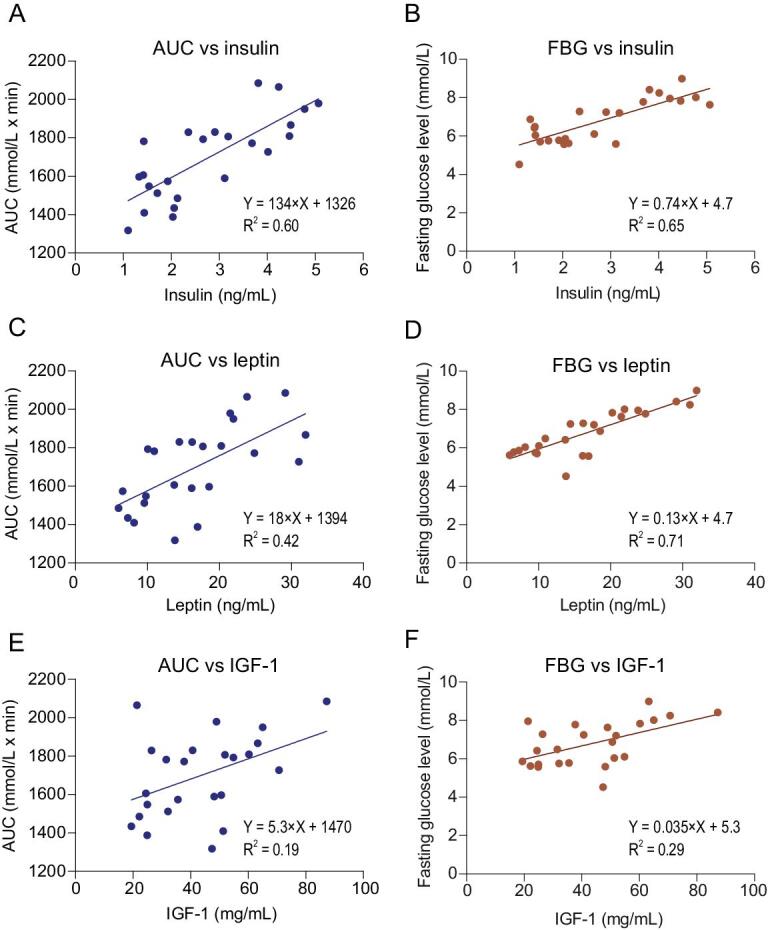
Linear regression between area under the glucose curve (AUC) or fasting blood glucose and blood hormone levels. Values were mean values for each diet group. (A, B) Insulin. (C, D) Leptin. (E, F) IGF-1. See also Fig. S6.

The dominant impact of the dietary macronutrients on glucose homeostasis were mediated by their impacts on body fatness, rather than via direct effects of the diets themselves. The impacts of expanding fat tissue may be mediated in part by hormones that are related to the levels of body fatness. We therefore turned our attention towards the roles played by hormones that are linked to body fatness in mediating this association. Fasting blood hormone levels, including insulin, leptin and IGF-1, were measured. All three hormones were significantly positively correlated to body fat mass (*P* = 3.59 × 10^−6^ for insulin, *P* = 3.09 × 10^−9^ for leptin, *P* = 0.001 for IGF-1) (Fig. 4C–E). Significant positive relationships were also observed between circulating hormone levels and FBG (F*_1, 22_* = 40.883, *P* = 1.96 × 10^−6^ for insulin; F*_1, 22_* = 53.132, *P* = 2.67 × 10^−7^ for leptin; F*_1, 22__ _*= 9.084, *P* = 0.0064 for IGF-1) and AUC (F*_1, 22_* = 33.296, *P* = 8.35 × 10^−6^ for insulin; F*_1, 22_* = 15.845, *P* = 6.30 × 10^−4^ for leptin; F*_1, 22__ _*= 5.184, *P* = 0.033 for IGF-1) (Fig. 5). These results suggest that the impact of body fatness on glucose homeostasis was at least partially mediated through these hormones. We also excluded the potential non-linear relationships by examining the patterns of residual variation against the hormones (Fig. S6). Homeostatic model assessment for insulin resistance (HOMA-IR) were also calculated for the 24 diets, and plotted against dietary fat content or body fat mass. The results were almost identical to that in Fig. 2C and D and Fig. 4 (data not shown).

GLM was then used to explore the associations between insulin, leptin, IGF-1 and FBG levels or AUC of the glucose curve. There were no interaction effects between insulin, leptin and IGF-1 (*P* > 0.05), and therefore we removed all the interaction terms. The revised GLMs (AUC-GLM2 and FBG-GLM2) showed that only insulin was significantly associated with AUC of the glucose curve (*P* = 0.001), and both insulin (*P* = 0.006) and leptin (*P* = 0.001) were significantly associated with FBG levels.

According to the above results, body fat mass was associated with glucose homeostasis potentially mediated partly by insulin and leptin levels. Therefore, an AUC-GLM (AUC-GLM3) was generated against body fat mass, insulin and leptin levels, to check if body fat mass has an additional effect on AUC apart from the effects via insulin and leptin. This analysis showed that body fat mass (*P* = 4.61 × 10^−6^) was still significantly associated with AUC, while insulin (*P* = 0.207) and leptin levels (*P* = 0.093) were no longer significant. This suggests that apart from an effect of body fat on glucose homeostasis mediated partly through insulin and leptin, body fat mass probably also significantly impacted glucose homeostasis through additional pathway(s). Similarly, an FBG-GLM (FBG-GLM3) was also generated against body fat mass, dietary fat content, insulin and leptin levels. The results showed that all terms were not significantly associated with FBG, suggesting no additional factors/pathways impacted FBG apart from body fat mass, dietary fat content, insulin and leptin levels.

We then extracted the residuals from AUC-GLM2 with insulin as co-variate and FBG-GLM2 with insulin and leptin as co-variates, respectively, to quantify the effect of body fat on the residuals. As body fat mass had a significant effect on both the AUC and FBG levels from previous analysis, we then plotted the residuals from both GLMs against body fat mass, respectively (Fig. S7). As shown in AUC-GLM3, body fat mass was still significantly associated with AUC, and it explained 9.4% of the variation of residuals from the AUC-GLM2 excluding the effect of insulin and leptin. Only 4.0% of the variation was explained by fat mass in the linear regression model against the residuals from the FBG-GLM2, as there were no additional effects of body fat on FBG from FBG-GLM3 (F*_1, 22_* = 0.92, *P* = 0.348).

### Expression of genes in the epididymal white adipose tissue and subcutaneous white adipose tissue correlated to the residuals from AUC-GLM and FBG-GLM

Transcriptomic analysis of both epididymal white adipose tissue (eWAT) and subcutaneous white adipose tissue (sWAT) revealed expression of 18,202 identified genes. We calculated the correlations between residuals from the GLM of either AUC or FBG against blood hormone levels and each of the genes. The results revealed 73 genes in eWAT whose gene expression levels were correlated with the residuals from the GLM with AUC against blood hormone levels, but no gene expression levels were correlated with the residuals from the GLM of FBG against blood hormone levels (for full list see Table S1). For the sWAT, there were 115 genes positively correlated to the residuals from the GLM with AUC against blood hormone levels (for full list see Table S1). Moreover, 14 of the 115 genes were positively correlated with the residuals from the GLM with FBG against blood hormone levels.

Adipose tissues secrete a large number of proteins (adipokines), which may play key roles in regulating glucose homeostasis in animals. We then assessed if the 73 positively correlated genes in the eWAT and 115 positively correlated genes in the sWAT encode proteins with a signal peptide, indicating that they may be secreted proteins. In the eWAT, 31 out of 73 genes encode signal peptides, while 27 out of 115 genes in the sWAT encode signal peptides. To further investigate the potential roles of these genes encoding signal peptides in glucose homeostasis, we then assessed the associations between each of these genes and the AUC of the glucose curve. The results showed that 2 of 31 genes in the eWAT and 11 of the 27 genes in the sWAT were directly and significantly associated with the AUC of the glucose curve.

Since both body fat mass and these genes potentially play important roles in both FBG levels and the AUC of the glucose curve, we explored the relationship between these genes and body fat mass. We then performed regression analysis between body fat mass and the identified genes encoding signal peptides (31 genes from eWAT and 27 genes from sWAT). There were 11 of 31 genes in the eWAT and 9 of 27 genes in the sWAT significantly associated with body fat mass.

**Figure 6.**
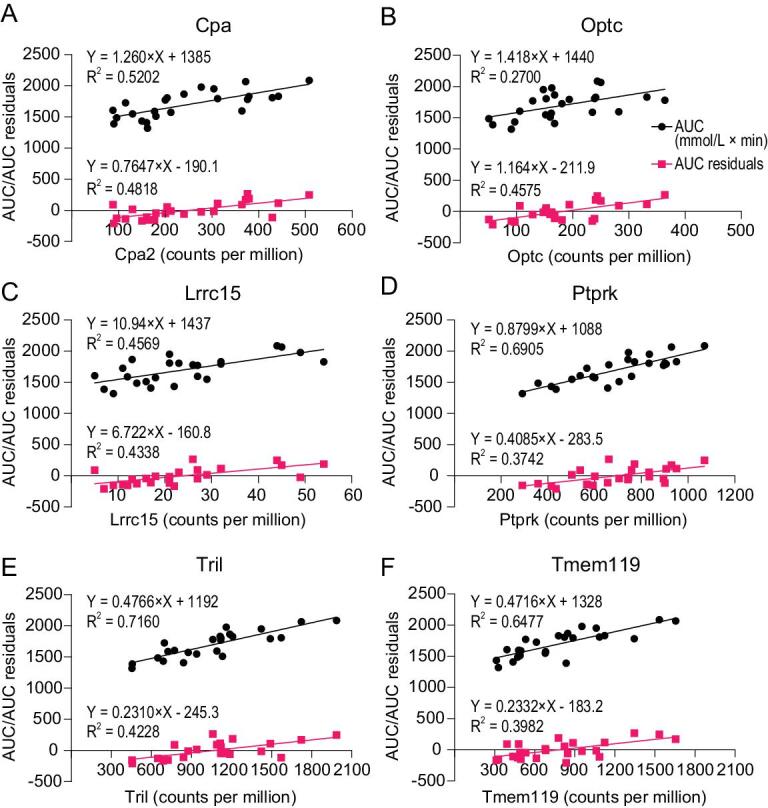
Linear regression between area under the glucose curve (AUC) and AUC residuals and the expression levels of six genes. Values were mean values for each diet group. (A) Cpa2. (B) Optc. (C) Lrrc15. (D) Ptprk. (E) Tril. (F) Tmem119.

We then compared the two genes from eWAT and 11 genes from the sWAT (which were significantly associated with the AUC of the glucose curve) with the 11 genes from the eWAT and nine genes from the sWAT (which were significantly associated with body fat mass). The results showed that one gene from eWAT and five genes from sWAT were significantly and directly associated with both body fat mass and the AUC of the glucose curve. These genes were Tmem119 (transmembrane protein 119), Cpa2 (carboxypeptidase A2), Optc (opticin), Lrrc15 (the type I transmembrane protein 15-leucine-rich repeat containing membrane protein), Tril (TLR4 interactor with leucine-rich repeats) and Ptprk (Protein tyrosine phosphatase receptor type kappa). The six genes were then regressed to AUC and AUC residuals from the GLM, respectively. Expression of all six genes showed a positive relationship with both AUC and AUC residuals (Fig. 6). Another GLM (AUC-GLM4) was then generated against body fat mass, insulin, leptin levels, plus the six gene expression levels. Expression of the Tmem119 gene was still significantly associated with AUC (*P* = 0.011), while body fat mass, insulin and leptin levels, and the other five gene expression levels were all not significant (*P* > 0.05). This shows that body fat mass may impact glucose homeostasis in mice through the effect of insulin, leptin and mechanisms related to these five genes. The effect of the five genes on glucose homeostasis may be part of the effect of body fat. Tmem119 may play a role on glucose homeostasis through an unknown pathway independent of body fat.

## DISCUSSION

We designed 29 diets varying orthogonally in their macronutrient compositions and exposed C57BL/6N mice to these diets for 12 weeks. We investigated the changes in body mass, fat mass and lean mass and their impacts on glucose homeostasis (FBG and AUC) after 10 weeks of diet intervention. Variation in protein levels (between 5% and 30%) and sucrose levels had no effect on both FBG levels and AUC. The main impact on FBG and AUC was due to body fatness which explained 38.8% of the variation in AUC and 38.6% of the variation in FBG. Dietary fat content explained an additional 1.5% of the variance in FBG. Fasting blood insulin, leptin and IGF-1 levels were all positively associated with body fat mass of the mice, and also positively associated with both FBG levels and AUC of the glucose curve.

Previous studies have suggested that carbohydrate intake may play a role in glucose homeostasis [15]. Glucose tolerance is a function of glucose stimulated insulin secretion, hepatic glucose output and tissue insulin sensitivity [25]. In rats, a HS (30% in solution) and high fat diet (HFD) induced glucose intolerance (both fasting glucose and AUC) after 13 weeks [16]. HF (45%) and HS (50%) diets caused different types of glucose intolerance in mice, probably due to peripheral insulin resistance in HF fed mice and reduced early insulin secretion in HS fed mice, respectively [17]. C57BL/6 mice fed an HFD (23.7% fat and 53.3% carbohydrate) developed glucose intolerance as early as after three weeks compared to a diet with 13% fat and 60% carbohydrate [18]. HF and low carbohydrate diets caused impaired glucose tolerance in mice [19] and rats [20]. The effect of dietary protein on glucose tolerance in animals has also been reported, yet the results were inconsistent [21–24]. In rats, 35% dietary protein and 20% fat impaired glucose tolerance probably due to the high level of saturated dietary fat [21], while high protein (52%) and low fat (9%) diet improved glucose tolerance [22]. However, a high (60%) protein and low carbohydrate diet significantly impaired glucose tolerance, and led to higher insulin levels and homeostatic model assessment (HOMA) values in mice after eight weeks feeding, in comparison with medium (33%)/low (5%) protein and medium/high carbohydrate diets, with fixed 20% fat in all three diets [23].

Most of these previous studies have focused on comparing only two or three diets. Moreover, in many cases the composition of these diets is not ideal for separating the impacts of different macronutrients because the compositions of the specific macronutrients are confounded by differences in other components. Furthermore, few studies have attempted to separate whether the impacts on glucose homeostasis stem from dietary effects or are mediated via impacts on body composition.

In our study increased dietary protein content did not lead to a decreased body fat mass, which differed from a previous study suggesting high protein diets improved glucose tolerance in rats due to reduced fat mass [22]. The discrepancy between studies may be the much higher levels of protein (52%) in their diets. Another large dietary manipulation study investigated 25 diets in 858 mice and reported some different outcomes from the present study [24]. In the previous study, high-protein diets reduced food intake when protein intake was over 10 kJ/day [24], in contrast to our diets where both food intake and energy intake were not changed when dietary protein content increased from 15% to 30% and protein intake was above 10 kJ/day. As we discussed previously, there was a difference in the range of the protein content, 5%–30% protein in our diets and 5%–60% in their diets [8,24]. In their study mice were fed with the experimental diets at three weeks old, while in our study mice were not exposed to the experimental diets until 10 weeks of age [8,24]. The metabolism of nutrients in mice during early life [26,27] and old age [24] is different from that which takes place during adult life. There might be an early life developmental effect of mice in their study, which was not discussed as the reported results were collected between six months and 15 months of age [24]. By contrast, in our study mice had the same background diets until they were switched to experimental diets at 10 weeks old. A GTT was performed at 15 months of age, which is late middle age, in their study [24], however, it was performed at about five months of age in our study. Overall, the two studies represent different nutritional metabolism of mice at different stages of their life.

Previously, HS diets were suggested to impair glucose tolerance in rats and mice [16,17]. Although we recently reported that higher liquid sucrose intake impaired glucose tolerance response in mice, this effect was more likely due to the higher body weight and body fatness than the higher absolute sucrose intake [28]. There might be differences between mice strains, mice and rat, sucrose delivery mode or even differences in the microbiome composition across animal housing conditions at different places. The mice developed glucose intolerance when fed on diets with high fat content, independent of carbohydrate content, consistent with previous studies [17–20]. These studies suggested that increased body mass, body fat mass and lean mass were likely related to the impaired glucose tolerance [17–20].

Blood insulin levels were associated with both FBG and AUC, while leptin was only associated with FBG levels. AUC-GLM3 showed a significant association between body fat mass and AUC, suggesting a significant additional effect of body fat mass on glucose tolerance apart from an effect potentially mediated via insulin and leptin, and body fat mass explained 9.4% of the residual effect from AUC-GLM2. This low percentage variation might be because that genes expressed in fat that are only loosely linked to the amount of fat mass. In longitudinal studies, higher fasting insulin levels reduced the subsequent body weight gain [29,30]. Also, it was reported that there was a causal effect of adiposity on increasing fasting insulin level [31]. Leptin levels were also previously correlated with body fat mass [32].

The six genes expressed in adipose tissue that we identified as linked to the AUC are likely to be potential genes that play important roles in glucose homeostasis, and may underpin the additional role of body fat mass in glucose control. None of the genes have been previously reported as potential genes mediating the direct link of obesity to diabetes. Importantly, Tmem119 [33] and Ptprk [34,35] are involved in TGF-β signaling. Blocking of the TGF-β signaling pathway induces browning of the white adipose tissue [36,37], and systematic blockade protects mice from obesity, diabetes and hepatic steatosis and thus may be a therapeutic strategy for obesity [36,37] and diabetes [36]. The TGF-β signaling pathway has also been associated with type 2 diabetes risk in genome-wide association studies (GWAS) [38,39]. Therefore, Tmem119 and Ptprk may play important roles in the development of obesity and diabetes. In addition, protein tyrosine phosphatases receptors have been associated with glucose homeostasis in mice (Ptprs and LAR) [40,41] and with type 2 diabetes risk in GWAS (Ptprd) [42]. Ptprd, Ptprs and LAR belong to R2A subtype, and Ptprk is the R2B subtype of PTP, which are very close subtypes [43], and thus Ptprk may also have the potential to impact glucose regulation. Cpa2 is one of the three carboxypeptidases secreted from the pancreas in the form of inactive precursors known as procarboxypeptidases [44]. Cpa2 is expressed in rat pancreas [45], and at extremely low abundance in the brain and several other extrapancreatic tissues such as the lung and testis (about four orders of magnitude lower than in the pancreas) [46]. Cpa2 has not previously been described from adipose tissue. It is a known secreted peptide which exerts carboxypeptidase activity preferentially at the 3’ end of target proteins. Lrrc15 [47], Tril [48] and Optc [49] all have leucine rich repeats. A recent paper suggested that leucine rich repeat domain proteins may interact with protein tyrosine phosphatase receptor type kappa (PTPRK) protein, which links to TGF-β signaling [50].

In conclusion, we observed a significant effect of body fat on glucose homeostasis by affecting both FBG levels and glucose clearance after a glucose challenge. Only dietary fat content impacted FBG levels, but not glucose clearance. As we have recently reported [9], dietary fat content regulates body fat mass in mice. Therefore, we propose that dietary fat content impacts glucose homeostasis in mice mainly through its effect on body fat mass, with a small direct effect on FBG levels. The association between body fat and glucose homeostasis may be partly mediated through blood insulin and leptin, as well as pathways linked to six identified genes in white adipose tissue. In particular, Tmem119 and Ptprk may play a role in TGF-β signaling.

## METHODS

### Ethical statement

All procedures were reviewed and approved by the Institutional Review Board, Institute of Genetics and Developmental Biology, Chinese Academy of Sciences (approval numbers AP2014011 and AP2016039).

### Mice and experimental diets

C57BL/6N male mice were purchased at age eight weeks from Charles River. The number of mice across all experiments was 300. The mice were exposed to 29 different diets with varying macronutrient contents (Table S2, which updates previous table [9]).

### Body mass, food intake and body composition measurements

Body mass and food intake were measured daily. Body composition including fat mass and lean mass were measured weekly using an EchoMRI^TM^ Body Composition Analyzer. Canola oil was used as the standard.

### Intraperitoneal glucose tolerance test

An ipGTT was performed 10 weeks after diet exposure. Following overnight fasting, glucose (2 g/kg body mass) was injected IP following measurement of fasting glucose. Blood (2–3 μL) was obtained by a tail prick at 15, 30, 60, 90 and 120 min, and glucose concentrations were measured using a glucometer. The total area under the glucose concentration-time curve (AUC) was calculated over 2 h following glucose injection.

### Blood hormone measurements

Following clotting, blood samples were centrifuged (3500 rpm, 30 min) to separate serum. Serum leptin, insulin and IGF-1 levels were determined using Mouse Leptin ELISA kit (90 030), Ultra-Sensitive Mouse Insulin ELISA kit (90 080) and Mouse IGF-1 ELISA kit (80 574) (Crystal Chem Inc., US), respectively. Five individuals were randomly chosen for each diet group, and mean values of each group were used for linear regression with body composition and AUC.

### RNA sequencing (epididymal and subcutaneous white adipose tissue)

From each diet group, the sWAT and eWAT of 12 individuals were collected. Each diet group had one pooled sWAT sample of six mice and one pooled eWAT sample of the other six mice. Total RNA was extracted using RNeasy Mini Kit (QIAGEN, 74 104). RNA sequencing was performed using the Illumina NextSeq 500 sequencer as reported [9]. Genes with counts per million (CPM) values ≥ 1 in at least one diet group were retained.

### Biological interpretation

The CPM values for genes from the eWAT and sWAT were correlated with the residuals of generalized linear models with AUC or FBG against hormone levels. The significant genes were then aligned with the Uniprot database to explore whether they encode signal proteins. Genes encoding a signal peptide were considered for further analysis. Step-wise regression and GLMs were performed between AUC and insulin levels and CPM values of genes encoding signal peptides. CPM values of these genes were then regressed to AUC and AUC residuals of the GLMs.

### Statistical analysis

Statistics were performed using IBM SPSS 20, GraphPad Prism 6.0 and Microsoft Excel. All values are expressed as mean ± SD. One-way ANOVA with Bonferroni post hoc testing was performed. Differences were considered significant if *P* < 0.05. The GLM was performed to relate FBG or AUC to body fatness and dietary macronutrients or hormone levels. Residuals were exported from the GLMs, and then associated with related parameters.

## Supplementary Material

nwaa177_Supplemental_File
