# Supplementary material for: Effects of dietary macronutrients and body composition on glucose homeostasis in mice
Source: Natl Sci Rev. 2020 Aug 6;8(1):nwaa177. doi: 10.1093/nsr/nwaa177 (PMC8288336; doi:10.1093/nsr/nwaa177)
Supplement: nwaa177_Supplemental_File [file nwaa177_supplemental_file.docx]

**Supplementary Data**

**Materials and Methods**

**Mice.** Upon arrival, C57BL/6N male mice were housed in groups to acclimate to the animal facility, before being separated at age 10 weeks into individual housing. All mice were then housed individually (in a specific pathogen free facility) and maintained in environmentally controlled conditions (temperature 22-24°C, 12: 12 h light/dark cycle, lights on at 07: 30). Appropriate housing temperature for studies of mice and how best to mimic the situation in humans is disputed. We used the temperature and cage conditions that were suggested by Speakman and Keiger (2012) as being appropriate [1]: temperature 23-25 ^o^C for single housed mice with bedding and nesting materials. They were provided *ad libitum* access to food and water and were monitored for health status daily. All mice were fed a standard diet with 10% fat and 20% protein (D12450B, Research Diets Ltd) for 2 weeks as the baseline period. Following 2 weeks of baseline monitoring (at age 12 weeks), all mice were randomly allocated to different groups and switched to the experimental diets for 12 weeks. After 12 weeks all mice were sacrificed and dissected.

**Experimental diets.** In total, mice were fed on 5 diet series, each series consisting of 6 different diets (total = 29 diets because 1 diet was common to two series). In the first two series (Series 1: D14071601 – D14071606 and series 2: D14071607 – D14071612) we fixed the level of fat at 20% (series 1) or 60% (series 2) by energy, and varied the protein content from 5% to 30% by energy. The protein source was casein. The balance was made up by carbohydrate (roughly equal mix of corn starch and maltodextrose). The diets within each of the two series were isocaloric, but differed between each series in energy density. The source of fat was a mix of cocoa butter, coconut oil, menhaden oil, palm oil and sunflower oil. This mix was designed to match the balance of saturated, mono-unsaturated and polyunsaturated fats (ratio 47.5: 36.8: 15.8) and the n-6: n-3 ratio (14.7: 1) in the typical western diet. The proportions of the different fat constituents and hence fatty acid distributions did not change as the total fat content changed. Sucrose and cellulose were both fixed 5% by energy and weight respectively, and all diets were supplemented with a standard vitamin and mineral mix. In the second two series of diets (series 3: D14071613 – D14071618 and series 4: D14071619 – D14071624) we fixed the level of protein at 10% (series 3) or 25% (series 4) by energy and then allowed the fat content to vary. When the protein was at 10% the six fat contents were 10, 30, 40 50 70 and 80%. When the protein was 25% the six fat contents were 8.3, 25, 33.3, 41.7, 58.3 and 66.6%. One consequence of varying fat content in this way is that the diets were not isocaloric – that is they did not have a constant energy density. This is an inevitable consequence of fat having a greater energy density than carbohydrate and protein. Hence, we cannot separate the impact of dietary fat content from a potential impact of energy density. This could be offset by adding an inert indigestible filler such as cellulose to maintain energy density constant, but then the fat content would covary with the level of the insert filler, and this would also dilute the other components. There is no way around these issues apart from being aware that when we infer an impact of fat this could be an impact of energy density. Since normally energy density is strongly correlated to fat content this may not in practice make any real difference. Fat, protein and carbohydrate composition were the same as those in the first 2 series. In these diets the sucrose, cellulose and vitamin and mineral contents were the same as the diets in series 1 and 2. In a fifth series of diets we fixed the fat at 41.7%, and the protein at 25% and then allowed the sucrose to vary between 5% and 30% in 5% steps (diet codes D16053101 to D16053105 plus diet D14071622 from series 4). All these diets can be ordered direct from research diets ([www.researchdiets.com](http://www.researchdiets.com)) using the diet codes provided.

**Intraperitoneal glucose tolerance test (ipGTT).** There is controversy about how to choose the glucose injection dose for such tests. Many studies have dosed in relation to body weight, but some authors have suggested the injection dose should be based on lean mass [2, 3]. Yet others suggest a fixed dose should be injected to all mice independent of body weight, as is performed in oral glucose tolerance tests in humans [4, 5]. The choice of which path to follow is not straightforward since injecting in relation to fat-free mass assumes only this compartment partakes in glucose disposal, which is known to be incorrect. But injecting in relation to body weight assumes both fat and lean compartments partake equally which is also incorrect. Consistent with many other studies, in the present study we injected glucose according to the body weight of each mouse, hence leaner mice were given a lower glucose dose. This would tend to favour finding an effect of lower body fat on the glucose tolerance as we did. Whether this is an artefact of the dosing protocol remains open for debate.

**RNA isolation and transcriptome analysis (epididymal and subcutaneous white adipose tissue (eWAT and sWAT)).** All sequencing of the RNA samples was carried out using the Illumina NextSeq 500 sequencer. RNA fragments were sequenced by 75 bp long reads from paired ends (PE 2x75 bp, 150 bp per fragment). FASTQ data files were analyzed using FASTQC (a quality control tool for high throughput sequence data; http://www.bioinformatics.babraham.ac.uk/projects/fastqc/). Paired-end reads were mapped to the *Mus musculus* genome (GRCm38) using Bowtie 2-2.1.0, Samtools-0.1.19, and TopHat-2.0.10; uniquely mapped reads for each gene were counted against the GTF file of GRCm38 provided by Ensembl (release 81) using HTSeq-0.6.1p1 using the strand = reverse; after obtaining the count data from the TopHat-HTSeq pipeline, genes with the counts per million (CPM) value ≥ 1 in at least one of the 24 diets group were retained [6].

**Biological Interpretation.** Since all the variables are continuous and there was no categorical variables, the residuals were extracted for further analysis. The cutoff point for the correlation between the CPM values of the genes and the residuals of GLMs were calculated using the random function in Excel. We generated randomized data and correlated the gene expression for each gene with these random data. We calculated the mean and standard deviations of the correlation coefficients between all 18202 genes and the random data, which were 0 and 0.1512, respectively. We then used the value of 4 times of the standard deviation 0.6049 as the cutoff point for the correlation coefficients in the real analysis. Genes with correlations beyond 0.6049 and -0.6049 were considered positively or negatively significant. Correlations of such magnitude would only occur with a probability of 0.005, if there was no actual correlation.

**Statistical analysis.** Based on the previously reported variation in the response of C57BL/6N mice to high fat diet [7], a power analysis indicated that to detect an effect size of 0.42 g/day in mean food intake between groups with 80% power at alpha = 0.05 in a one-way ANOVA with 6 levels, a sample of 10 per group was necessary, while to detect an effect size of 0.76 g/day a sample of 5 per group was necessary. A total of 240 male C57BL/6N mice were therefore used to investigate the effect of protein and fat content on adiposity, at 10 mice per diet. The power of fasting blood glucose levels (FBG) and area under the curve of the glucose curve (AUC) from the study were also calculated using a one-ANOVA with 6 levels and sample size of 10. The power of blood glucose levels was 71.6% with the maximum difference of 3.4 and standard deviation of 2.2, while the power of the AUC was 65.4% with the maximum difference of 745.8 and standard deviation of 513.2.

**References:**

1. Speakman, J.R. and Keijer, J., *Not so hot: Optimal housing temperatures for mice to mimic the thermal environment of humans.* Mol Metab, 2012. **2**(1): p. 5-9.

2. Ayala, J.E., Samuel, V.T., Morton, G.J., et al., *Standard operating procedures for describing and performing metabolic tests of glucose homeostasis in mice.* Dis Model Mech, 2010. **3**(9-10): p. 525-34.

3. McGuinness, O.P., Ayala, J.E., Laughlin, M.R., et al., *NIH experiment in centralized mouse phenotyping: the Vanderbilt experience and recommendations for evaluating glucose homeostasis in the mouse.* Am J Physiol Endocrinol Metab, 2009. **297**(4): p. E849-55.

4. Heikkinen, S., Argmann, C.A., Champy, M.F., et al., *Evaluation of glucose homeostasis.* Curr Protoc Mol Biol, 2007. **Chapter 29**: p. Unit 29B.3.

5. Muniyappa, R., Lee, S., Chen, H., et al., *Current approaches for assessing insulin sensitivity and resistance in vivo: advantages, limitations, and appropriate usage.* Am J Physiol Endocrinol Metab, 2008. **294**(1): p. E15-26.

6. Anders, S., McCarthy, D.J., Chen, Y., et al., *Count-based differential expression analysis of RNA sequencing data using R and Bioconductor.* Nat Protoc, 2013. **8**(9): p. 1765-86.

7. Zhang, L.N., Morgan, D.G., Clapham, J.C., et al., *Factors predicting nongenetic variability in body weight gain induced by a high-fat diet in inbred C57BL/6J mice.* Obesity (Silver Spring), 2012. **20**(6): p. 1179-88.

**Supplemental Figures:**

**
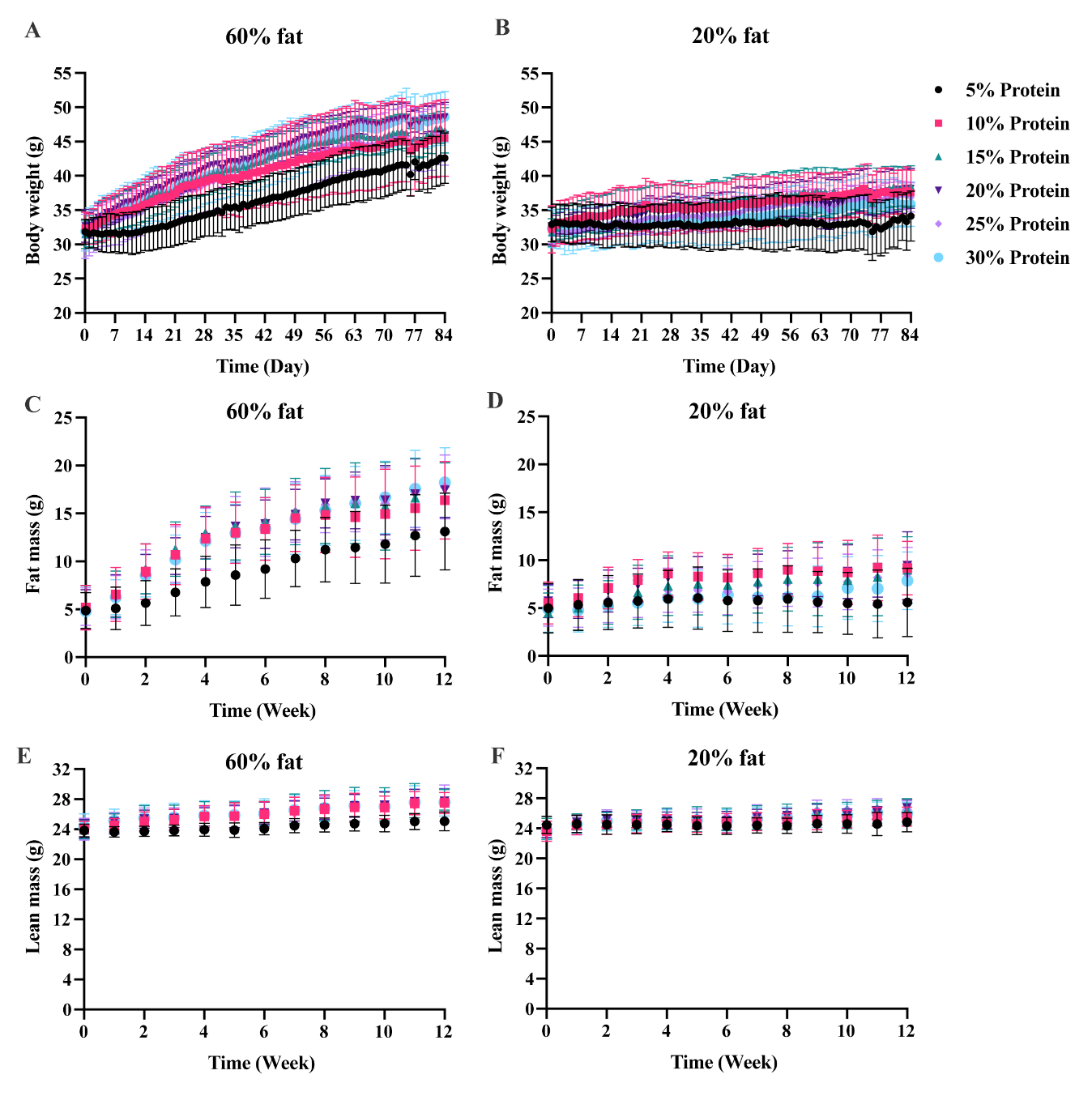
**

Figure S1 Body composition of the mice fed on diets with fixed 60% or 20% fat and variant protein content. Body weight of the mice on diets with 60% fat (A) or 20% fat (B). Body fat mass of the mice on diets with 60% fat (C) or 20% fat (D). Body lean mass of the mice on diets with 60% fat (E) or 20% fat (F).

**
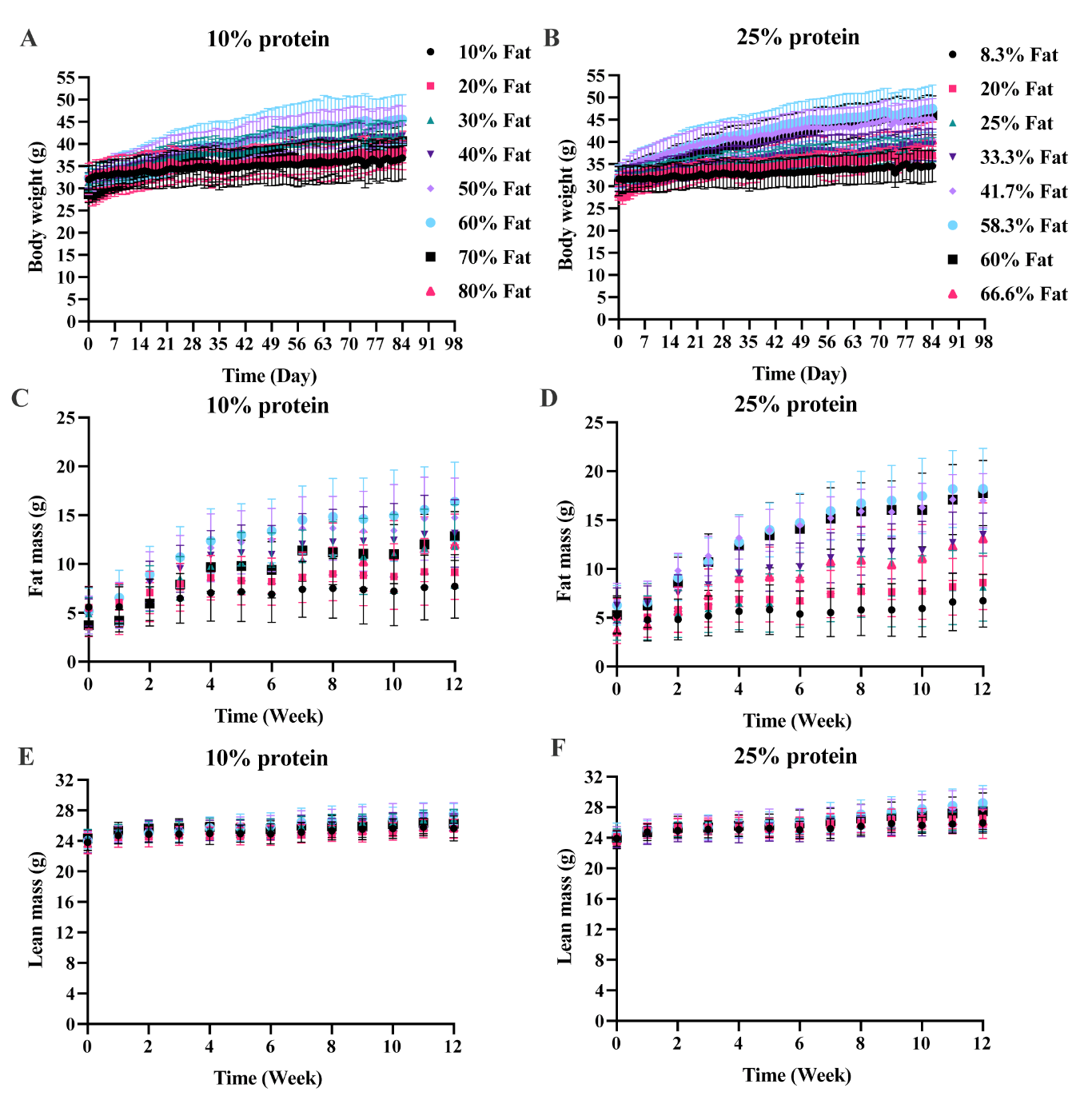
**

Figure S2 Body composition of the mice fed on diets with fixed 10 or 25% protein and variant fat content. Body weight of the mice on diets with 10% protein (A) or 25% protein (B). Body fat mass of the mice on diets with 60% fat (C) or 20% fat (D). Body lean mass of the mice on diets with 60% fat (E) or 20% fat (F).

**
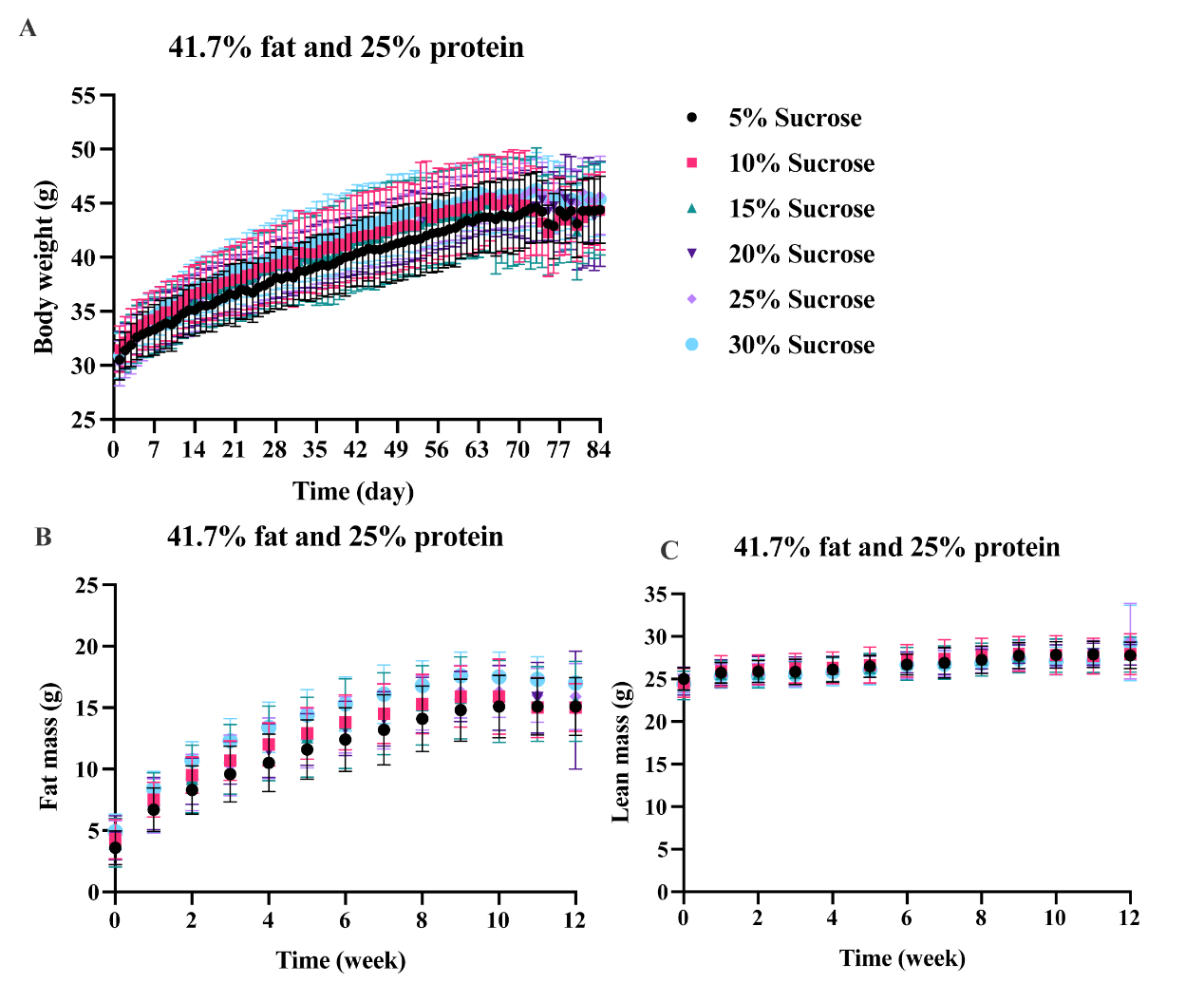
**Figure S3 Body composition of the mice fed on diets with fixed 41.7% fat and 25% protein and variant sucrose content. (A) Body weight. (B) Body fat mass. (C) Body lean mass.


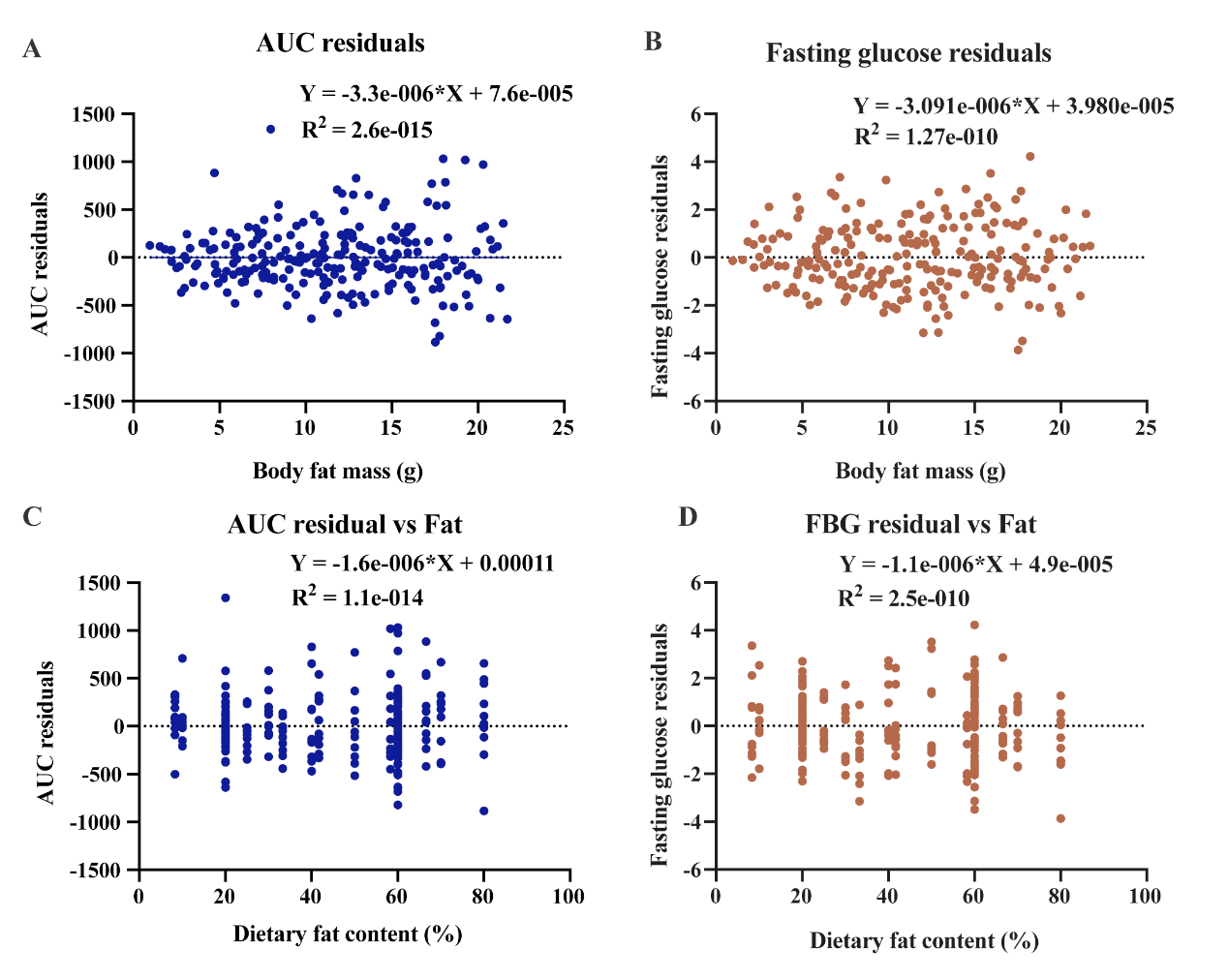
Figure S4 Linear regression between AUC-GLM residuals or fasting blood glucose-GLM residuals and (A-B) Body fat mass. (C-D) Dietary fat content.


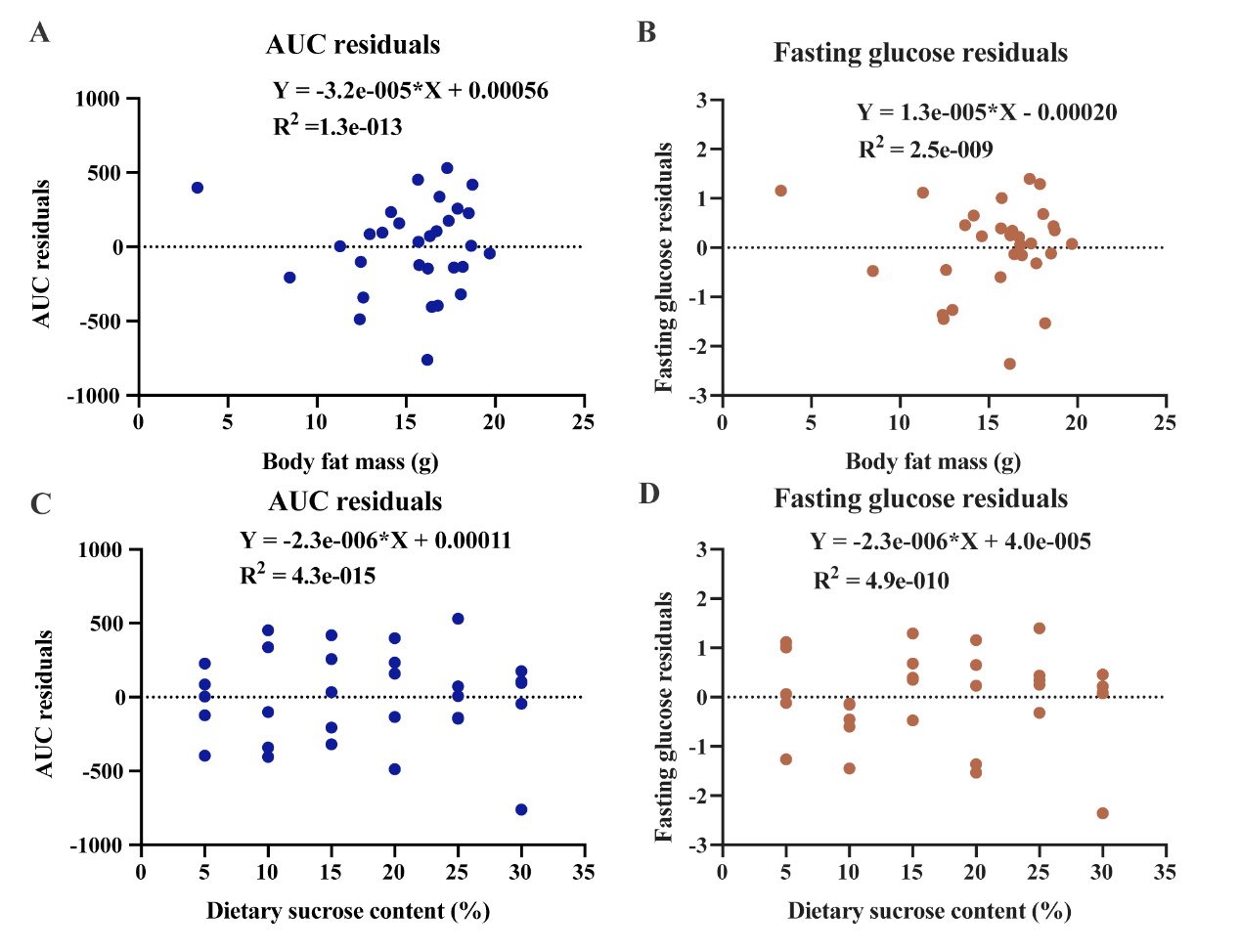
Figure S5 Linear regression between AUC-GLM residuals or fasting blood glucose-GLM residuals and body fat mass or dietary sucrose content. (A-B) Body fat mass. (C-D) Dietary sucrose content.


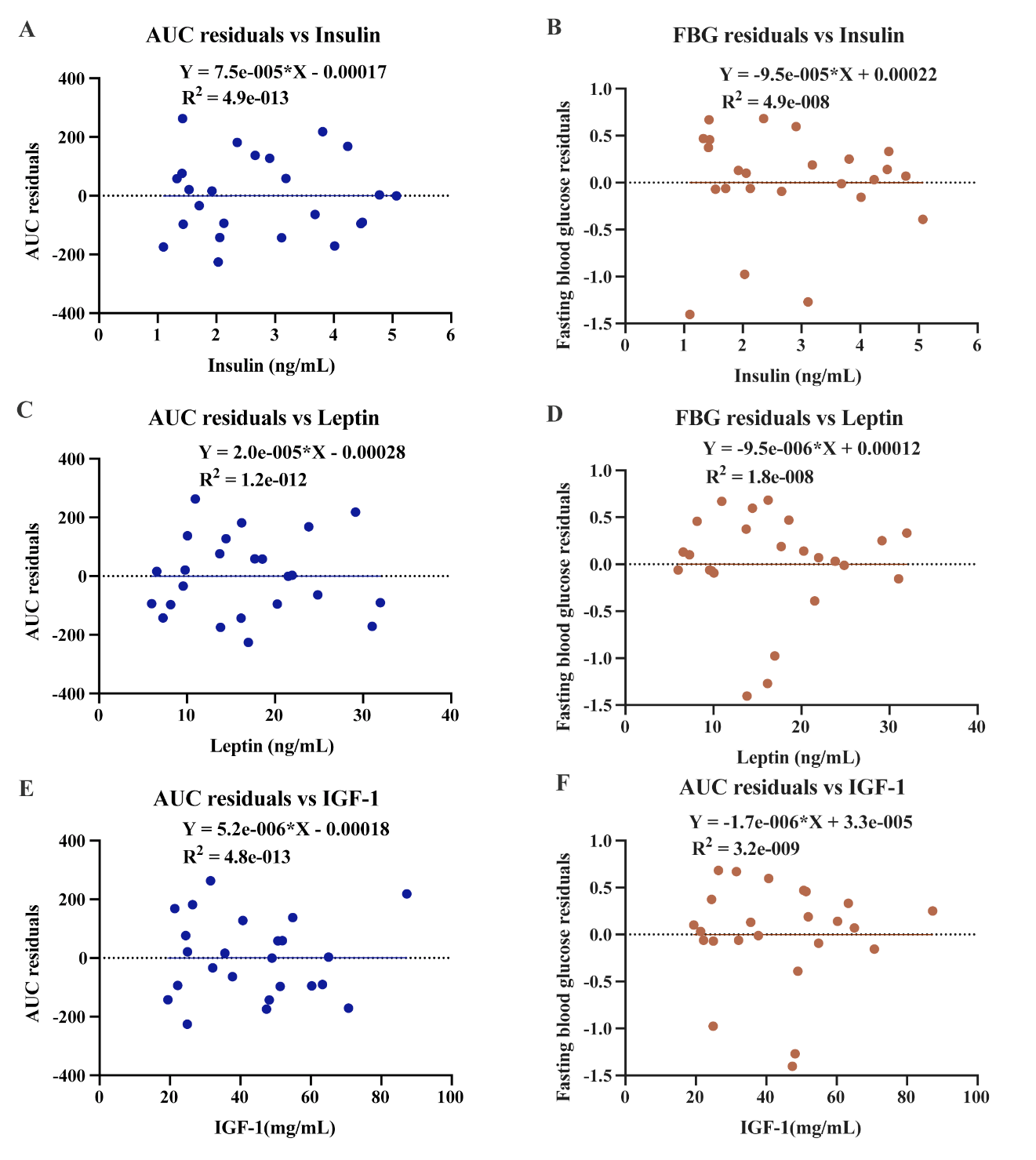
Figure S6 Linear regression between AUC-GLM residuals or fasting blood glucose-GLM residuals and blood hormones. (A-B) Insulin. (C-D) Leptin. (E-F) IGF-1. Related to Figure 6.


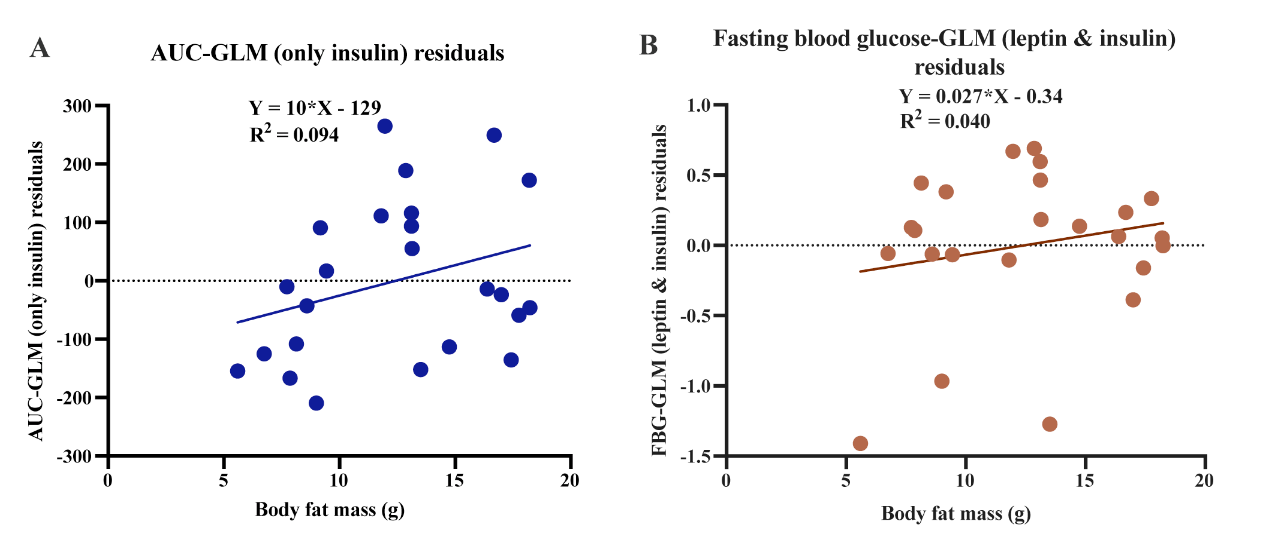
Figure S7 Linear regression between AUC-GLM residuals (A) or fasting blood glucose-GLM residuals (B) and body fat mass.

**Supplemental table titles and legends:**

Table S1 The significantly correlated genes with area under the curve (AUC) and fasting blood glucose in the sWAT and eWAT.

Table S2 Composition of the 29 experimental diets.
